# Myocardial infarction with normal coronary arteries: a case report and review of the literature

**DOI:** 10.1186/1752-1947-3-24

**Published:** 2009-01-23

**Authors:** Luigi Iuliano, Fausta Micheletta, Alessandro Napoli, Carlo Catalano

**Affiliations:** 1 Department of Internal Medicine, Unit of Vascular Medicine, Sapienza University of Rome, Corso della Repubblica, 04100 Latina, Rome, Italy; 2 Department of Radiological Sciences, Sapienza University of Rome, via del Policlinico 155. 00161 Rome, Italy

## Abstract

**Introduction:**

Although acute myocardial infarction is generally associated with obstructive coronary artery disease, myocardial infarction associated with normal coronary arteries is a well-known condition. The overall prevalence rate of myocardial infarction with normal coronary arteries is considered to be low, varying from 1% to12% depending on the definition of "normal" coronary arteries.

**Case presentation:**

We describe here a case of a 49-year-old woman with a history of prior myocardial infarction who continued to be asymptomatic after a 10-year follow-up, in the absence of a high-risk profile for adverse outcomes. She was studied with multi-slice coronary computed tomography and whole-body angiography, which showed normal coronary and extra-coronary arteries.

**Conclusion:**

This case report raises two important issues. First, the possible role of multi-slice computed tomography/coronary angiography in the risk- and prognosis assessment of patients with known or suspected coronary artery disease. Second, the important role played by long-term pharmacological therapy in patients with prior myocardial infarction and normal coronary arteries.

## Introduction

Myocardial infarction (MI) with normal coronary arteries is a medical condition, which has been described in the literature for more than 30 years but is still a challenge in medical practice because of the lack of evidence-based medical data on its prognosis and on secondary prevention. The prevalence of MI with normal coronary arteries has always been considered low, varying between 1% and 12%. However, it actually involves a considerable number of patients, calling for more clinical trials which specifically study this sub-population of patients affected by coronary heart disease (CHD) [1-3].

We report a case of a young female patient with a previous clinically diagnosed MI, who was asymptomatic in standard care therapy for a 10-year follow-up and has been shown to have normal coronary arteries by coronary multi-slice computed tomography (MSCT) and whole-body angiography.

## Case presentation

A 49-year-old woman was referred to our department for a cardiological follow-up visit. She had a medical history of hypertension and previous MI, with no history of diabetes mellitus, hypercholesterolemia or premature coronary artery disease in her family. She did not smoke, take recreational drugs and was not on oestrogen replacement therapy.

Ten years earlier, she had an inferior MI treated with systemic thrombolysis, unfractioned heparin, aspirin, atenolol and intravenous nitroglycerine. During her hospitalization, the echocardiogram (ECG) revealed akinesis of the posterior-basal wall with an estimated ejection fraction of 50%. Laboratory tests including serum glucose, lipids, blood count, liver-, kidney- and thyroid-function tests were within normal limits. The day before her discharge, she had a stress ECG negative for inducible ischemia. The patient was discharged on the sixth day with a drug regimen of aspirin, atenolol, captopril, simvastatin and isosorbide dinitrate.

After the MI and prior to the visit to our department, the patient had been free from chest pain and dyspnea and had a normal exercise tolerance. She had periodical ECGs that showed the presence of akinesis of the posterior-basal wall with a mildly depressed systolic function. She reported a good control of arterial blood pressure with the ongoing therapy, including perindopril 5 mg/day, hydroclhorotyazide 25 mg/day and atenolol 25 mg/day associated with aspirin and simvastatin 20 mg/day.

A physical examination showed blood pressure of 135/80 mmHg, heart rate of 64 bpm, BMI of 27 kg/m2, waist circumference of 85 cm and no findings of congestive heart failure. The resting ECG showed a sinus rhythm at 62 bpm and the presence of Q waves in II, III and aVF leads with no other abnormalities.

Laboratory tests revealed a total serum cholesterol of 160 mg/dl, HDL cholesterol of 55 mg/dl, triglycerides of 78 mg/dl, LDL cholesterol of 89.6 mg/dl and fasting glycemia of 116 mg/dl, with a normal OGTT and normal plasma homocysteine.

On the basis of her medical records concerning the MI, we confirmed the diagnosis of coronary artery disease (CAD) according to the recently published universal definition of MI [4].

For risk stratification, we studied the patient with non-invasive diagnostic tests, including echocardiography and a treadmill exercise test.

The ECG confirmed the presence of akinesis of the posterior-basal wall with a mildly depressed systolic function (LVEF 48%) and the treadmill exercise test showed no signs of inducible ischemia.

Without any symptoms which suggest cardiac ischemia, the results from the non-invasive tests suggested no high-risk criteria for adverse outcomes for our patient. According to the guidelines by the American Heart Association/American College of Cardiology (AHA/ACC), there was thus no indication for conventional coronary angiography.

Consequently, we decided to study her coronary arteries non-invasively. MSCT angiography was performed using a 64-slice computed tomography (CT) scanner (Sensation Cardiac 64; Siemens; Forchheim, Germany). The protocol included a post-coronary phase for whole-body angiography.

**Figure 1:**
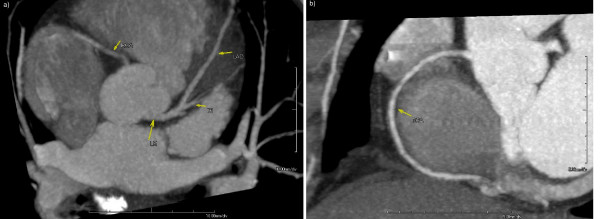
**Coronary computed tomography angiography shows absence of flow limiting stenosis in both left (1a) and right (1b) coronary circulation**. A tri-dimensional data set was reformatted using the maximum intensity projection technique. In Figure 1a, the image was taken along the anterior interventricular plane, allowing fine analysis of the left main as well as the left anterior descending coronary artery. A maximum intensity projection view also allows partial assessment of the origin of the right coronary artery and the first diagonal branch. Figure 1b: an image acquired along the right atrio-ventricular groove; the maximum intensity projection image clearly depicts the right circumflex artery up to the inferior portion.

**Figure 2:**
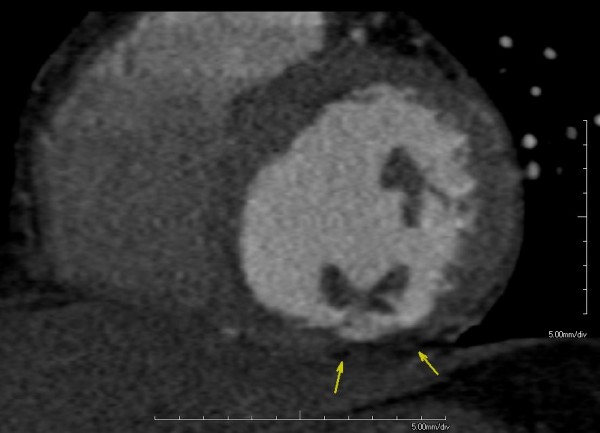
**Multiplanar reformatted images generated along the conventional short cardiac axis, showing focal areas of myocardial wall thinning associated with hypodensity (arrows), suggestive for non-recent myocardial wall infarction**.

The MSCT angiographic analysis showed no coronary lesions (Figure 1), but the presence of a hypodense area involving more than 50% of the myocardial wall of the left ventricular inferior basal portion (Figure 2). No lesions were detected in the wholly explored extra-coronary arterial system (not shown).

The patient was sent to follow-up with a drug regimen of perindopril 5 mg/day and hydrochlorothiazide 25 mg/day. Aspirin, atenolol and simvastatin were discontinued. She was advised to lose weight and implement some lifestyle changes and was asked to repeat laboratory tests for a lipid profile before the next follow-up visit.

At the 6- and 12-month follow-up, the patient was free from chest pain, LDL-cholesterol was below 140 mg/dl without statin therapy and blood pressure was stable below 140/90 mmHg.

## Discussion

The overall prevalence rate of MI with normal coronary arteries is considered to be low, varying from 1% to 12%, depending on the definition of "normal" coronary arteries, which usually includes no luminal irregularities (strict definition) or arteries with some degree of stenosis (in most cases, less than 30% stenosis) [1-3]. The incidence seems to be strongly related to age and sex, with higher rates in young and female patients [5]. Thus, studies of women less then 45 years old, who have suffered acute MI, have showed normal coronary arteries angiographically in 7% to 32% of cases [1,5,6]. We have described a case of a young female patient with a history of prior MI who, after a 10-year follow-up, continued to be asymptomatic with no high-risk profile for adverse outcomes. She was studied by MSCT angiography, which showed normal coronary arteries.

This case report raises two important issues. First, the possible role of MSCT coronary angiography in risk- and prognosis assessment of patients with known or suspected CAD. Second, the role of long-term pharmacological therapy in patients with prior MI and normal coronary arteries.

MSCT provides high-resolution coronary angiograms non-invasively and has been demonstrated to be highly accurate in ruling out coronary atherosclerosis [7]. Its high negative predictive value for exclusion of significant coronary artery stenosis, approaching 100%, makes it an attractive method for the assessment of patients with known or suspected CAD and no high-risk profile for adverse outcomes; patients for whom conventional coronary angiography is not indicated according to current international guidelines. In particular, it might have an important application in patients for whom a CAD diagnosis was made on a clinical basis with no angiographic documentation.

We approached our patient according to a diagnostic algorithm based on the current AHA/ACC guidelines. Our patient had been asymptomatic since the MI event and had had no high-risk features for adverse outcomes in routine non-invasive tests. Medical history, clinical features and non-invasive tests did not provide specific diagnosis and prognosis, which allowed us only to continue the current medical therapy. According to the scheme provided by the current guidelines, we should have considered further imaging studies. However, in our patient, routine non-invasive imaging studies, such as nuclear stress testing or stress echocardiography would not have provided any new information for a better risk- and prognosis stratification. We considered the low age and the low cardiovascular risk profile of the patient at the time of acute MI, the absence of symptoms after the event and the absence of inducible ischaemia during the treadmill exercise test and decided that a non-invasive delineation of the presence and severity of coronary atherosclerosis would allow insights for the further management of our patient.

MSCT angiography showed absolutely normal coronary arteries. The absence of atherosclerosis lesions in this patient was further confirmed in the extra-coronary arterial tree, thus providing a definitive diagnosis. The high negative predictive value of MSCT coronary angiography allowed us to definitely exclude CAD and the need for further diagnostic tests. Moreover, MSCT provided a more accurate prognosis for our patient for two main reasons. First, long-term prognosis in patients with MI and normal coronary arteries is much better compared to patients with coronary occlusive disease, especially in young and female patients [2,5,8]. Patients with normal coronary arteries have a good survival rate, around 90% at 3–7 years of follow-up in different studies, and a significant lower rate of reinfarction than patients with obstructive coronary disease [2,5,8]. Second, it has been recently demonstrated that MSCT provides independent prognostic information on baseline clinical risk factors in patients with known or suspected CAD, showing an excellent prognosis in patients with normal coronary arteries [9].

The diagnosis of prior MI with normal coronary arteries raises a second important issue concerning the application of AHA/ACC secondary prevention guidelines for CHD. According to these guidelines, patients who have had MI should receive indefinitely a drug regimen including an antiplatelet agent, a beta-blocker, a statin with an LDL-C goal of < 100 mg/dl or < 70 mg/dl, and an ace-inhibitor with a blood pressure goal < 140/90 mmHg [10]. As stated in the paper, cases covered by these guidelines include patients with established coronary and other atherosclerotic vascular disease. Thus, cases with prior MI and normal coronary arteries are actually not covered by these guidelines, and there are no other indications on the management of these patients in the literature.

The estimated annual incidence of MI, new and recurrent in the US is 865 000, and among these, about 350 000 are women (11). Among these women, the above-mentioned 7% to 32% with normal coronary angiography translate into 24 500 to 112 000 women with acute MI and normal coronary arteries annually in the US alone. This sub-population of patients affected by CHD is significant and the lack of randomized clinical trials, comparing therapies for the reduction of adverse cardiac events in patients with MI and normal coronary arteries, makes their management challenging. It is difficult to find a physio-pathological rationale that allows us to transfer secondary prevention guidelines from patients with established coronary artery disease to patients with normal coronary arteries.

On the other hand, these patients are young people with good prognoses, which implies that a long-term multi-drug therapy should be carefully considered in terms of cost-benefit analysis. Despite an undemonstrated improvement of the prognosis, a multi-drug regimen including antiplatelet agents, statins, beta-blockers and ace-inhibitors would expose our patient to well-known adverse effects and imply a considerable economic cost.

According to the clinical features and the diagnostic test results from our patient, we decided to retain the ace-inhibitor because of arterial hypertension and to discontinue aspirin, statin and beta-blocker because of the absence of coronary atherosclerotic disease, hypercholesterolemia and symptoms reflecting inducible ischaemia.

## Conclusion

MSCT coronary angiography can have an important role in the risk and prognosis assessment of patients with known or suspected CHD. Large-scale randomized trials need to be conducted to determine an optimal secondary prevention strategy for patients with MI and normal coronary arteries, who constitute a large sub-population of CHD patients.

## Abbreviations

AHA/ACC: American Heart Association/American College of Cardiology; CAD: coronary artery disease; CHD: coronary heart disease; CT: computed tomography; ECG: echocardiogram; MI: myocardial infarction; MSCT: multi-slice computed tomography; OGTT: oral glucose tolerance test.

## Consent

Written informed consent was obtained from the patient for publication of this case report and accompanying images. A copy of the written consent is available for review by the Editor-in-Chief of this journal.

## Competing interests

The authors declare that they have no competing interests.

## Authors' contributions

LI was in charge of the patient clinically and was a major contributor in writing the manuscript. MF collected clinical data and reviewed the literature. AN prepared a CT angiography protocol to study in a single-setting coronary and whole-body arterial system. CC analyzed and interpreted the imaging studies. All authors read and approved the final manuscript.
